# The Need of a Trained Microscopist in Every Community1Read at the thirty-ninth annual meeting of the Medical Association of Central New York, at Syracuse, October 16, 1906.

**Published:** 1907-07

**Authors:** William C. Krauss

**Affiliations:** Buffalo, N. Y.


### BUFFALO MEDICAL JOURNAL.

VOL. LXII. JULY, 1907. NO. 12

### ORIGINAL COMMUNICATIONS.

### The Need of a Trained Microscopist in Every Community.[1]

BY WILLIAM C. KRAUSS, M.D. Buffalo, N. Y.

THE early trend of civilisation westward was marked by a sequence of events which was characteristic of the early settlers and pioneers, and which might well be called the *evolution* of *society.*

The pioneer in casting around for a settlement, generally chose a clearing near some running stream of water. In course of time, a run of stone, driven by water power, crude but efficient for the purpose, was the first indication that a stand was to be made for the development of a community, or settlement. The anvil and forge became a most necessary adjunct, and soon the blacksmith took his place at one of the corners of the cross roads. The influx of strangers and the urgency of harboring them comfortably, necessitated a suitable place of refuge and the tavern arose on one of the other corners. The merchant soon took his place at the remaining corner with his general store, including the post office, and which in due course of time served as club house, meeting place and a general clearing house for the exchange of opinion as well as of produce. Thus was established the miller, tavernkeeper, blacksmith and the merchant, the four pillars upon which modern society was to be erected. The log-cabin school was not long in building, neither was the meeting-house, which hand in hand, gave the growing settlement a dash of ethical as well as of spiritual culture. Events followed quickly, the ravages of disease, the growing population and the demand for operative treatment decided the physician to make his domicile here with the rest.

Prosperity and population increased hand in hand with rapid strides and the railroad took its place with the other elements

1. Read at the thirty-ninth annual meeting of the Medical Association of Central New York, at Syracuse, October 16, 1906.

of civilisation. The four corners had evolved from a hamlet to a village and was fast entering upon the threshold of a city. The merchant, the preacher, the physician and the teacher became the four pillars, around whom the ethical life of the community revolved. As the life of the community became more complex, the duties of the professional man augmented also, but to none more so than the general practitioner, who, until now, had served as physician, surgeon, obstetrician, alienist, and in short an all around general specialist. Division of medical work by segmentation gave rise to the surgeon, the ophthalmalogist and in short the general specialist, one who knew little of many things and nothing much of any one thing. Still he professed his knowledge, and his faults and vices served to attract others who *were* efficient and well trained in their chosen fields of special study. The hospital was the natural sequence of events and the bringing together of the better qualified and stronger elements in the profession. With it, the trained nurse appeared and to all appearances, the plant had a modern, scientific foothold. But as hospital work progressed, and frequent consultation and interchange of opinion followed, the literature of the day discussed, societies organised for mutual self-help and improvement, it soon became apparent that the highest scientific methods had not yet been developed, and the best results had not yet been attained. The determination of albumin and sugar was no longer sufficient for a urine content examination. The physical signs of diphtheria, typhoid and tuberculosis were too slow in developing to give the best results in the treatment of these diseases. The appearance of the blood meant very little by the old method. Medicolegal questions were constantly arising and no one in the community had the time, nor the mental training or equipment to connect the laboratory with the bedside and interpret quickly and correctly the phenomena presented by the ever changing chemistry of the body. The diagnosis of disease by guesswork or "rule of thumb" had had its day, and the patient demanded, as well as the community, that the most modern methods and the most modern procedures be adopted in the diagnosis and treatment of disease. Prevention of disease by a proper and scientific examination of the food and water supply, the proper regulation of the tenement house evil, general sanitary and hygienic reforms demanded the services of an expert clinicopathologist, one who understands his microscope as thoroughly as the surveyor his transit. Such a man must of necessity be the finished product of the laboratory, skilled in histological, pathological and bacteriological technique and the practical adaptability of the same to the necessities of the municipality. As a factor in the community, he can render inestimable service. He can detect the bacillus of tuberculosis long before the physical signs and symptoms are apparent. He can render a differential diagnosis early, between the Klebs-Loeffler bacillus of diphtheria and the ordinary streptococci of a simple tonsilitis. He can make a Widal test long before the diagnosis of typhoid fever is permissible. He can detect the source and nature of purulent discharges occurring about the body. In regard to the examination of the urinary contents, he is now able to delve deep into the chemical and histological mysteries and detect acetone, indican, diacetic and hippuric acids. and the end products of pathological changes. Along the alimentary canal, his work is of far reaching importance. The chemistry of the stomach is like an open book and he can read its mysteries with *his* test tube and lens as readily as the scholar reads his classics with *his* glasses. The cause and origin of the diarrheas become apparent and hence easily combated. The disturbances of assimilation are thoroughly ascertained and the deficiency or excessive flow of digestive secretion at once determined. The whole science of hematology, including blood pressure, is at his command, and the blood diseases are now more accurately diagnosed than any other class of ailments. With the advancement of diagnostic methods, sooner or later, therapeutic progress must also follow. This has already begun in reference to the leucocytosis in connection with the inflammatory processes of the closed cavities and operative measures have reduced mortality rates to a considerable degree.

In an interesting article on—"Of what Service is the Clinical Pathologist to the Clinician," Dr. Frederick E. Sondern, of Brooklyn, says:[2] Routine examinations of urine, blood, gastric contents, feces and some other secretions and excretions of the body are well within the scope of the clinician, provided he has the requisite time, the technical skill and maintains the necessary apparatus and reagents in efficient working order. The more complex chemical problems of the urine and other excretions such as the nitrogen partition, the sulphate partition, many of the more difficult quantitative determinations, can scarcely be undertaken by him with the hope of trustworthy results, and are better referred to the clinical pathologist. The main objection to the analytical work done by the clinician as a class, is not lack of accuracy, but rather a want of methodical procedure leading to complete investigation. The clinician is very apt to conclude his diagnosis and then make his urine analysis or blood examination with the purpose in mind, chiefly, to corroborate his clinical findings rather than with a view of excluding other possible conditions,

2. Long Island Medical Journal, March, 1907.

some of which may not occur to him at the time. A few examples may emphasise this point. A pallid young woman may show the clinical evidence of a chlorosis and her medical adviser, mindful of laboratory corroborating diagnostic aids, but lacking methodical procedure, determines the amount of hemoglobin to learn the degree of enemia, and test the urine for reaction and gravity as a matter of routine, and for albumin to exclude a possible renal lesion. Had both the blood and the urine examinations been complete he might have found a beginning leukemia, a slight diabetes, or a secondary enemia, instead of a chlorosis, with relative eosinophilia to justify a suspicion of possible intestinal parasites. One may argue that these other conditions give you quite a different clinical picture and that in diabetes the specific gravity of the urine would have attracted attention to the possible presence of glucose. True, ordinarily the clinical picture is indicative of the disease beyond question, but unfortunately there are exceptions.[2]

Again, ordinarily the gravity of diabetic urine is high, but from time to time you find glucose with a gravity of 1008, and these are just the cases with a typical clinical picture, in which you may ascribe the frequency of micturition to a neurotic polyura often seen in chlorotic girls. Allow me to picture another example. A patient in previous good health is confined to bed with an acute febrile disease, the exact nature of which has not yet become apparent. His physician, we will assume, looks upon malaria or typhoid as the most probable explanation, and may give him a few good doses of quinine before deciding on a Widal test and a plasmodia hunt. Widal reactions are tardy in appearing, and most varieties of plasmodia soon leave the peripheral circulation after the administration of quinine. While a complete blood examination in this case does not give you the diagnosis, it does narrow down the possibilities, and usually leads to an earlier recognition of the disorder. The presence of a leucocytosis and relative polynuculear increase, at once suggests a most probable inflammatory lesion, though not indicating its site, and at the same time lends information as to its virulence and the degree of body resistance offered. The absence of these signs allows the exclusion of the diseases which occasion them and thus suggest the possibility of the different febrile disorders which occur without them. While you seldom obtain a Widal reaction in the first few days of a typhoid, the very frequent leucopenia and relative lymphocytosis are suggestive and while plasmodia of malaria may, for one or other reason, not be found in that disease the decided increase in the relative number of large lymphocytes is usually a corroborating feature. Examples of this kind are easily multiplied and the lesson they teach is, that methodical and complete investigation of your specimens is necessary if you desire all the information they are capable of giving, and do not wish to occasionally meet with disagreeable surprises which such thoroughness might have avoided. In order to obtain the maximum information from laboratory aids in diagnosis with a minimum degree of error, a number of essentials must be carried in mind. Those of us who have argued in favor of one or other laboratory aid have always met with the response that such aid does not take the place of bedside observation. Allow me to say with all the emphasis of which I am capable, that no laboratory procedure, no matter how important, ever takes the place of clinical observation, and it is the man who is the most acute bedside observer that gets the most help from laboratory aids, for he is generally also well informed as to the value and significance of the changes from the normal noted in the laboratory observation. As usual, one encounters the two extremes, the young man overenthusiastic about laboratory help and apt to misinterpret it on account of neglect of bedside observation and the old one not sufficiently versed in the help to be had from laboratory aids or their significance and overconfident in his clinical intuition, both err and err badly sometimes, and justify the motto, "He who makes his diagnosis in the laboratory is as short sighted and liable to grave error as the man who ignores the microscope and the test tube." The diagnostician who strives to excel is the one who develops his bedside skill to the greatest possible degree, and who learns to apply the results of complete laboratory investigations as an aid in his work."

These are but some of the fields in which the clinico-pathologist is working. These are but a few of the problems which he will be called upon to solve. He will become the center, yes, and the head of the board of health and upon him, more than upon any other member of the Board will the hygienic prosperity of the community depend. No city or village of any importance can afford to be without the services of such a man and concerted effort of the profession with the city or town councils will find the means and appurtenances for the installation of a properly equipped and efficient clinicopathological laboratory. What can be done along the lines suggested can best be inferred by quoting at some length from the last Biennial Report of the Board of Health of Chicago.[3]

To those who have learned to look to Chicago as the one city where things are done upon a large and thorough scale, the last Annual Report of the Department of Health, will come as no

3 Editorial in the Cleveland Medical Journal, April, 1906.

surprise. To some of us, however, we are confident that it will be at least news to be told that Chicago is the healthiest city in proportion to the population in the country, and the mass of interesting and valuable data given in this report, only confirms the common impression of the attention given to detail and of the ability to cope with things on a large scale so commonly attributed to the Windy City. Chicago's Commissioner of Health is indeed to be congratulated upon the extraordinary showing made in this report, a showing so remarkable that we venture to quote from it at length.

DURATION OF LIFE IN CHICAGO.

One's attention is at once caught by the table published at the beginning of this report, setting forth graphically, the proportion of deaths at given ages to the total deaths at all ages in Chicago from 1875 to the close of 1905. The average age of decedents in Chicago during the year 1875 was 16 years, two months, and 12 days; during 1885, it had increased to 20 years, 4 months, and 26 days; during 1895, there was a further increase in the span of the average life to 24 years, 7 months, and 9 days; and during the year 1905, the average age of the decedents had reached 31 years and 10 months, almost double the average duration of life 30 years ago.

Even more interesting are the figures given in detail for the various year periods during this time, especially so being the figures of the proportion of deaths at a given age to the total deaths at all ages. During 1875 for those under one year, the percentage was 38.3; during 1905 this had been reduced to 21.4; during the period from two to five years, the figures given are 8.3% for 1875, as against 4.3% during 1905.

Just as the average percentage during the early age periods has diminished, so has the age period, in proportion to all ages increased in the periods above 30, until in the age period over 70, the figures given are 3.8% during 1875 as against 10.1% during 1905. Thus it will be seen that measured by the average age of all who died in the city of Chicago 30 years ago and all those who died in 1905, the average duration of life in this city has nearly doubled during a single generation.

CHICAGO'S ACTUAL DEATH RATE.

Up to November 22, 1905, the actual death rate in Chicago was 13.75 in 1000; the second lowest, we are told, in the city's history; the lowest annual death rate in Chicago's history occurring during the year 1904, when it was 13.62. This death rate is the lowest of any large city in the world with a population of 300,000 or over. As indicating the continued tendency to a lower death rate during the last 20 years, the following figures are of interest. In 1884, with a population of 629,885, the deaths numbered 12,471; in 1904, with a population of 1,932,315, the deaths numbered 26,311, an increase of only 13,840, not much more than twice as many, while the population was three times as great. The death rate in 1884 in Chicago was 19.63; in 1904 it was 13.62 per 1000. The following graphic percentages show this extraordinary betterment.

| | |
|---|---|
| Percent. of increase in population in 20 years, 1884-1904 . . . . . . . | 209.9 |
| Percent. of increase in actual number of deaths in 20 years . . . . . | 110.9 |
| Percent. reduction in the annual death rate in 20 years . . . . . . . . | 30.6 |

These figures mean that during the year 1904, owing to the improvement in Chicago's health, there was a saving of 11,620 lives as against the mortality which would have occurred had the death rate of 1884 prevailed during that year.

The actual death rate for the year 1905 was 13.67 per 1000, a rate 6.4% lower than that of Cleveland; 20.8% lower than that of Philadelphia; 25.4% lower than that of New York; and 26.1% lower than that of Boston. This record is truly extraordinary. The actual figures compiled from the reports furnished by the respective cities are given below. In this connection it is but fair to note that Chicago is the only one of these cities using the United States Census Office estimate of population, the other cities, excepting Boston, computing their death rate on an estimate of population much in excess of the Census Office estimate.

SOME DISEASES THAT HAVE BEEN CONTROLLED.

Interesting as showing the value of efficient sanitary administration, the greatest reductions of mortality have been shown among the diseases over which the Health Department have had the greatest and most direct control. Among the diseases which have thus shown a marked diminution not only in prevalence but in mortality rate, may be mentioned first small-pox. In the decade from 1885 to 1894 this scourge caused 1,072 deaths, a mortality rate of 0.83 per 10,000 of population, while during the last ten years, there were but 247 deaths or a mortality rate of 0.16 per 10,000 of population.

Diphtheria and croup which in the early decade carried off 13,566 victims, were responsible for only 8,168 deaths during the last 10 years. A mortality rate of 14.33 in the first period as against 5.2 in the second. A reduction of 63.7% and a constructive saving of 14,545 lives.

Even more interesting is the reduction of the mortality rate from typhoid fever and the diarrheal diseases. In the first decade typhoid fever caused 7,844 deaths, a mortality rate of 7.7 per 10,000 of population; in the second decade 5,392 deaths, a rate of 3.3 and a reduction of 57.1%. Had the death rate of the early decade prevailed, there would have been 7,348 more deaths from typhoid fever during the last 10 years.

Diarrheal diseases were responsible for 25,235 deaths in the early decade, with a death rate of 25.6% per 10,000 of population, while during the last decade there were but 24,165 deaths from this cause, with a mortality rate of 15. This reduction of 41.4% implies a saving of 18,193 lives; such results speak for themselves.

THE REDUCTION IN THE INCIDENCE OF CONTAGIOUS DISEASES.

It must be conceded that the decrease in the occurrence of measles, scarlet fever and whooping cough is due to improved hygienic conditions as well as better nutrition and care. The mortality for measles during the first decade was 1.91, in the second 0.83, a decrease of 56.6%. Scarlet fever has shown a decrease in its mortality rate of 55.5%, while whooping cough shows a decrease of 28.3%, a constructive saving for these three diseases of 4,887 lives by their reduced mortality.

THE CHIEF CAUSES OF DEATH FOR HALF A CENTURY.

Perhaps the most striking table which appears in this valuable report is that demonstrating graphically by means of colored disks, the 12 chief causes of death in each decade during the period from 1856 to 1905 together with their mortality rate for each 10,000 population during each decade. During the first decade 1856 to 1865, the 12 chief causes of death are given in the following order, diarrheal diseases, consumption, diphtheria and croup, scarlet fever, nervous diseases, violence, typhoid fever, pneumonia, small-pox, measles, malaria, and heart diseases. Comparing the fifth decade 1896 to 1905 with this period of 40 to 50 years together, one is struck by the great diminution in the mortality rate for every specific disease with the single exception of that from pneumonia. That the mortality rate from pneumonia should have increased is undoubtedly explainable by reason of its occurrence in those individuals whose life has already been prolonged by the increase in the average duration. During the last decade, pneumonia has ranked as the first cause of death and consumption has at no time fallen below the third cause of death. Curiously enough, the deaths from violence have ranked as the fifth or sixth cause throughout all this period. Diphtheria has fallen from the third chief cause of death to the tenth. Typhoid fever from the seventh to the 11th and scarlet fever from the fourth to the 12th, while small-pox has fallen below the first 12 causes of death. The figures given in this table are extremely interesting and will bear careful and critical analysis, showing clearly the results accomplished by modern hygiene and sanitation.

What are the agencies which have accomplished this remarkable results? Dr. Chas. J. Whalen, the Commissioner of Health, from whose summary report the above figures are taken, tells us that this has been accomplished by constant supervision of the water supply, with publicity of its daily condition, regulation of lake dumping, securing sewage diversion from the lake, the correction of more than 100 local defects in tunnels and pumping stations in a single year, the promotion of the drainage channel, and by a vast amount of work in the way of chemical and biological examinations.

The instructive report of the Chicago Board of Health can be duplicated to some degree by that of any of the larger progressive American cities. Notably—New York, Detroit, Milwaukee, Buffalo and many others and serves as a beacon to the smaller cities and villages which unfortunately are not so thoroughly and efficiently equipped for fighting disease.

A trained microscopist with a suitable laboratory is within the grasp of any community containing ten or more active-progressive physicians—if they will act in concert and induce the town or county authorities to help defray the expenses of such a plant.

The action of the Canandaigua (N. Y.) physicians is an example of what persistence, energy and push will do. Led by Drs. Beahan and Hallenbeck, members of this association, they have built up a hospital system, installed a clinico-pathological laboratory and publish monthly a very creditable report of the work they are accomplishing indicated by this paper.

Other communities can do the same thing and must do it—if they are to be representatives of the active scientific spirit—so rampant just at the present time.

479 DELAWARE AVENUE.

